# Bioactive Isopimarane Diterpenes from the Fungus, *Epicoccum* sp. HS-1, Associated with *Apostichopus japonicus*

**DOI:** 10.3390/md13031124

**Published:** 2015-03-02

**Authors:** Xuekui Xia, Jun Qi, Yayue Liu, Airong Jia, Yonggang Zhang, Changheng Liu, Cuiling Gao, Zhigang She

**Affiliations:** 1 Key Laboratory for Applied Microbiology of Shandong Province, Biotechnology Center of Shandong Academy of Sciences, Jinan 250014, China; E-Mails: xiaxk@sdas.org (X.X.); qljbgreat@163.com (J.Q.); jiaar@sdas.org (A.J.); zhangyg@sdas.org (Y.Z.); 2 School of Chemistry and Chemical Engineering, Sun Yat-Sen University, Guangzhou 510275, China; E-Mail: liuyayue@mail2.sysu.edu.cn; 3 Shandong Provincial Key Laboratory of Test Technology for Material Chemical Safety, Jinan 250014, China

**Keywords:** *Epicoccum* sp., secondary metabolites, isopimarane diterpene, X-ray diffraction, α-glucosidase inhibitory activity

## Abstract

One new isopimarane diterpene (**1**), together with two known compounds, 11-deoxydiaporthein A (**2**) and iso-pimara-8(14),15-diene (**3**) were isolated from the culture of *Epicoccum* sp., which was associated with *Apostichopus japonicus.* Their structures were determined by the analysis of 1D and 2D NMR, as well as mass spectroscopic data. The absolute configuration of Compound **1** was deduced by a single-crystal X-ray diffraction experiment using CuKα radiation. In the bioactivity assay, both Compounds **1** and **2** exhibited α-glucosidase inhibitory activity with IC_50_ values of 4.6 ± 0.1 and 11.9 ± 0.4 μM, respectively. This was the first report on isopimarane diterpenes with α-glucosidase inhibitory activity.

## 1. Introduction

The incidence and prevalence of type 2 diabetes, representing over 90% of all case of diabetes, are increasing rapidly as time passes. The International Diabetes Federation predicted that the number of people with diabetes would rise from 266 million in 2011 to 552 million by 2030 [1]. Alpha-glucosidase is a key enzyme for breaking down carbohydrates for absorption [2]. Agents inhibiting α-glucosidase are effective therapeutic agents for diabetes [3]. The development of new drugs for use against α-glucosidase is therefore urgently needed.

Marine fungi have attracted more attention for their good bioactivity against α-glucosidase [4,5,6]. In the course of our continuing search for biologically-active substances from fungi derived from mangrove and sea cucumber [7,8,9], we had screened extracts from a number of fungi, and those with interesting biological activities were routinely subjected to chemical exploration. The ethyl acetate extract from the fungus, *Epicoccum* sp. HS-1, associated with *Apostichopus japonicus*, showed α-glucosidase inhibition activity. One new isopimarane diterpene (**1**), together with two known compounds, 11-deoxydiaporthein A (**2**) and isopimara-8(14),15-diene (**3**), were isolated from the extract. To date, biomedical and pharmaceutical studies of isopimarane diterpene have shown interesting activities, such as cytotoxicity [10,11], acetylcholinesterase inhibitory activity [12] and anti-HIV-1 bioactivity [13]. To our knowledge, there have been no previous reports of the α-glucosidase inhibition activity of these. The details of the isolation, structural elucidation and the results of the α-glucosidase inhibition study of the isolated compounds are reported herein.

## 2. Results and Discussion

### 2.1. Chemical Structure Elucidation

Compound **1** was isolated as colorless crystals. The molecular formula was established as C_21_H_30_O_6_ on the basis of HR-ESI-TOF/MS data for *m/z* 401.1938 [M + Na]^+^ with seven degrees of unsaturation. Twenty seven protons were bound to carbons, and three exchangeable hydrogens were present. Detailed analysis of the ^1^H, ^13^C, HMQC, as well as DEPT spectra of Compound **1** revealed the presence of one methoxy group at δ_H_ 3.23/57.6 (21-CH_3_O), two oxygenated methylene groups at δ_H_ 4.43 and 3.22/69.8 (H-20a, H-20b), two oxygenated methine groups at δ_H_ 4.42/ 62.8 (H-11) and δ_H_ 4.15/80.0 (H-14), three methyl groups at δ_H_ 0.81/25.5 (CH_3_-17), δ_H_ 1.18/28.1 (CH_3_-19) and δ_H_ 1.51/23.8 (CH_3_-18), sp^3^ methylene groups at δ_H_ 2.15 and 1.67/23.3 (H-1a, H-1b), δ_H_ 1.82/17.8 (H-2), δ_H_ 1.60 and 1.28/37.1 (H-3a, H-3b), as well as δ_H_ 2.30 and 1.78/40.8 (H-12a, H-12b), an sp^2^ methylene group at δ_H_ 5.07 and 5.11/112.3 (H-16a, H-16b) and an sp^2^ methine group at δ_H_ 6.20/145.3 (H-15). In addition, five sp^3^ and two sp^2^ non-hydrogenated carbon signals appeared at δ_C_ 36.8, 41.2, 52.9, 81.2, 105.0, 133.2, 165.7, as well as a carbonyl group at 193.4. On the basis of these data, Compound **1** was hypothesized to have a diterpene skeleton.

**Figure 1:**
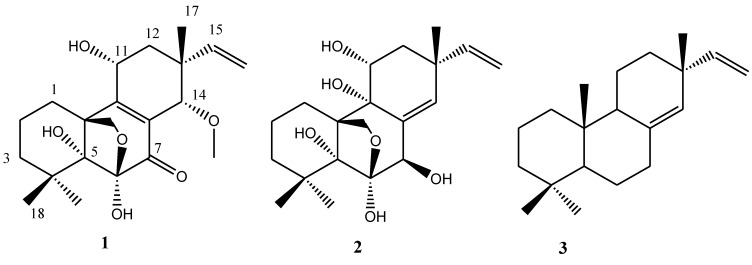
Structures of isolated Compounds **1**–**3**.

Compound **1** was also presumed to have four rings in its structure based on the unsaturation degree, since it contained two double bonds and a carbonyl group. The COSY spectrum of Compound **1** revealed the presence of three spin systems, including CH_2_(1)CH_2_(2)CH_2_(3), CH(11)CH_2_(12) and CH(15)CH_2_(16). The extension of the spin systems and attachments of functional groups were confirmed by HMBC correlations. The HMBC correlations of CH_3_-18 and CH_3_-19 to C-4 and C-5 indicated that CH_3_-18 and CH_3_-19 were attached to C-4 at δ_C_ 36.8, CH_3_-17 to C-13, C-14 and C-15, H-15 to C-13 and C-16, indicating that both CH_3_-17 and CH (15) = CH_2_ (16) were attached to C-13, and 21-OCH_3_ to C-14, suggesting that OCH_3_ was linked to C-14. H-20a with C-6 in HMBC allowed the linkage of oxygenated an sp^3^ methylene carbon C-20 at δ_C_ 69.8 to the hemi-acetyl carbon C-6 at δ_C_ 105.0 through oxygen to form a dihydrofuran ring. Ultimately, the chemical structure of Compound **1** was identified as shown in Figure 1. Detailed ^1^H and ^13^C NMR data are presented in Table 1. More detailed spectra are available at the Supplementary Figures S1–S7.

**Table 1:** ^1^H (MeOD, 600 MHz) and ^13^C (MeOD, 150 MHz) NMR data of Compound **1 **^a^.

Position	δ_C_, mult.	δ_H_	HMBC (H→C)
1	23.3, CH_2_	a, 2.15 (dt, 4.8, 13.8); b, 1.67 (m)	C-2, 10
2	17.8, CH_2_	1.82 (2H, dt, 6.0,13.0)	C-1, 3
3	37.1, CH_2_	a, 1.60 (dt, 3.0, 13.8); b, 1.28 (m)	C-2, 5
4	36.8, C		
5	81.2, C		
6	105.0, C		
7	193.4, C		
8	133.2, C		
9	165.7, C		
10	52.9, C		
11	62.8, CH	4.42 (dd, 2.0, 8.4)	C-8, 9, 12
12	40.8, CH_2_	a, 2.30 (dd, 2.0, 14.4); b, 1.78 (dd, 9.0, 14.4)	C-9, 11, 13, 15
13	41.2, C		
14	80.0, CH	4.15 (s)	C-7, 9, 21-OCH_3_
15	145.3, CH	6.20 (dd, 11.4, 18.6)	C-16
16	112.3, CH_2_	a, 5.07 (d, 12.0); b, 5.11 (d, 18.0)	C-14, 15
17	25.5, CH_3_	0.81 (s)	C-13, 14, 15
18	23.8, CH_3_	1.51 (s)	C-4, 5, 19
19	28.1, CH_3_	1.18 (s)	C-4, 5, 18
20	69.8, CH_2_	a, 4.43 (d, 9.0); b, 3.22 (d, 9.0)	C-5, 6, 9
21	57.6, CH_3_	3.23 (s)	C-14

^a^ δ in ppm, *J* in Hz.

The relative configuration of Compound **1** was established on the basis of NOESY correlations of H-20a (δ 4.43, d, *J* = 9.0 Hz)/CH_3_-18 (δ 1.51, s)/H-11 (δ 4.42, dd, *J* = 2.0, 8.4 Hz) and CH_3_-17 (δ 0.81, s)/H-14 (δ 4.15, s). The coupling constants derived from coupling between H-11 (δ 4.42, dd, *J* = 2.0, 8.4 Hz), H-12a (δ 2.30, t, *J* = 2.0, 14.4 Hz) and H-12b (δ 1.78, t, *J* = 9.0, 14.4 Hz) confirmed the equatorial position of H-11.

**Figure 2:**
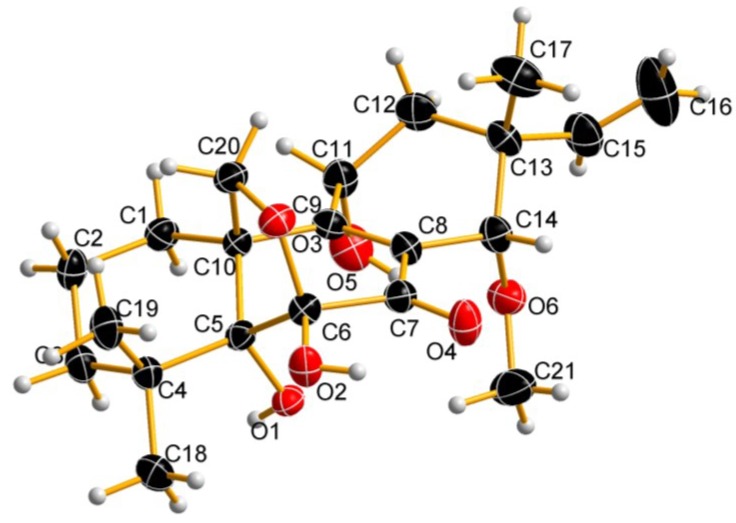
Perspective oak ridge thermal ellipsoid plot (ORTEP) drawing of Compound **1**.

The absolute configuration of **1** was further confirmed by a single-crystal X-ray diffraction experiment using CuKα radiation (Figure 2) with Cambridge Crystallographic Data Centre (CCDC) No. 1000803, which was defined as 5*R*, 6*S*, 10*S*, 11*R*, 13*R* and 14*S*.

The structure of Compound **2** was identified as 11-deoxydiaporthein A by comparison of its NMR, MS and CD data with those available in the literature [14]. Compound **2** showed a positive Cotton effect at 217 nm, which was similar to that of 11-deoxydiaporthein A isolated from marine fungus, *Cryptosphaeria eunomia* var*. eunomia* [14], and showed only mild activity against *Mycobacterium tuberculosis* with a MIC value of 200 μg/mL [15]. Compound **3** was identified as *ent*-pimara-8(14),15-diene by comparison of its NMR and MS data [16], which was named as isopimara-8(14),15-diene in this paper. Isopimara-8(14),15-diene was obtained from engineered *Aspergillus nidulans* and showed significantly higher DPPH radical scavenging activity than beta-carotene [16].

### 2.2. Biological Activity

All compounds were tested for their *in vitro* inhibitory activities against α-glucosidase according to a previously described method with modifications [17]. The results are listed in Table 2. Compounds **1** and **2** exhibited more effective inhibitory activities, with IC_50_ values of 4.6 ± 0.1 and 11.9 ± 0.4 μM, respectively, than resveratrol, which was used as the positive control, while Compound **3** did not exhibit inhibitory activities (IC_50_ > 200 μM) in this test (Table 2).

**Table 2:** Inhibitory effects of the isolates against α-glucosidase.

Compounds	IC_50_ (μM)
**1**	4.6 ± 0.1
**2**	11.9 ± 0.4
**3**	>200.0
Resveratrol ^a^	31.2 ± 4.4

^a^ Positive control.

## 3. Experimental Section

### 3.1. General Experimental Procedures

Optical rotations were measured on an Anton Paar MCP 300 polarimeter (Ashland, VA, USA) at 30 °C. UV data were recorded on a Shimadzu UV-240 spectrophotometer. CD spectra were recorded on a JASCO J-815 spectropolarimeter using a Nicolet Magna-IR 750 spectrophotometer. ^1^H and ^13^C NMR spectra were recorded on a Bruker AVANCE 600 (600 and 150 MHz). Chemical shifts were reported in δ (ppm), using tetramethylsilane (TMS) as the internal standard, and coupling constants (*J*) were in Hertz (Hz). HR-ESI-TOF/MS was performed on an Agilent 6530 high-resolution mass spectrometer. Single-crystal data were measured on an Agilent Gemini Ultra diffractometer (CuKα radiation). Column chromatography (CC) was performed on silica gel (200–300 mesh, Qingdao Marine Chemical Factory, Qingdao, China) and a Sephadex LH-20 (Amersham Pharmacia Biotech AB, Stockholm, Sweden). Precoated silica gel plates (G60, F-254, Yan Tai Zi Fu Chemical Group Co., Yantai, China) were used for thin-layer chromatography. Semipreparative HPLC was performed on a Waters HPLC system consisting of a 2695 pump, a 2998 PDA (potato dextrose agar), an autosampler and a YMC C_18_ column (250 × 10 mm, 5 μm); flow rate: 2.0 mL/min. Compounds **1**–**3** were isolated as the secondary metabolites from cultures of the *Apostichopus japonicus*-derived fungus strain, *Epicoccum* sp. HS-1. Resveratrol was purchased from Adamas-beta Ltd. Co. (Shanghai, China).

### 3.2. Fungal Material

The HS-1 strain [8] was cultured on slants of PDA at 25 °C for 7 days. Agar plugs were cut into small pieces (1-cm diameter each) under aseptic conditions, and 10 pieces were used to inoculate into five Erlenmeyer flasks (500 mL), each containing 100 mL of media (glucose 10 g/L, peptone 2 g/L, yeast extract 1 g/L and crude sea salt 3 g/L). Five flasks of inoculated media were incubated at 25 °C on a rotary shaker (170 r.p.m.) for seven days to prepare the seed culture. The mycelium was aseptically transferred to 100 Erlenmeyer flasks (500 mL) containing a total of 20 L of liquid medium consisting of glucose (10 g/L), peptone (2 g/L), yeast extract (1 g/L) and crude sea salt (3 g/L), then incubated at room temperature for 28 days.

### 3.3. Extraction and Isolation

The fermentation medium was filtered through cheesecloth. The filtrate was concentrated below 50 °C to a final volume of 5 L and extracted three times with ethyl acetate (1:1, v/v), and the extracts were pooled and concentrated to dryness with a rotary evaporator at 45 °C. The resulting crude extract (5.2 g) was subjected to vacuum liquid chromatography (VLC) with silica gel (60, <0.663 mm, Merck, Darmstadt, Germany). The column system was sequentially eluted by 500 mL of each of the following solvents: petroleum, petroleum/ethyl acetate (9:1, 4:1, 7:3, 1:1, 1:4, v/v); ethyl acetate, ethyl acetate/MeOH (4:1, 7:3, 1:1, 1:4, 0:5 v/v). At last, 10 fractions were obtained, all of which were monitored by thin liquid chromatography (TLC) in 254 and 310 nm. Fraction 2 was further purified by a Sephadex LH-20 using CH_2_Cl_2_/MeOH (1:1, v/v) to obtain Compound **3** (14.0 mg). Fraction 5 (30.6 mg) was separated on a Sephadex LH-20 using CH_2_Cl_2_/MeOH (1:1, v/v) to give Compound **2** (7.1 mg). Fraction 4 (21.0 mg) was purified by a Sephadex LH-20 using CH_2_Cl_2_/MeOH (1:1, v/v) to give ten subfractions (Fraction 4-1–4-10), and Fraction 4-5 and Fraction 4-6 were combined and then subjected to high performance liquid chromatography (HPLC) over octadecylsilane (ODS) (YMC, 250 × 10 mm) (30% MeOH-H_2_O, v/v, 5 min; 30%–100% MeOH-H_2_O, v/v, 45 min; 100% MeOH, 55 min ) to give Compound **1** (retention time (*t*_R_) 38.4 min; 4.4 mg).

Isopimarane diterpene (**1**): colorless crystals. UV (MeOH): λ_max_ (log ε): 191 (0.62) and 248 (0.31) nm. CD (MeOH) λ_max_ (θ in mdeg) 191.2 (38.1), 193.9 (−4.85), 250.0 (−3.68), 266.7 (−5.03); [α]^30^_D_ −242. (*c* 0.024 MeOH); HR-ESI-TOF/MS: *m/z* 401.1938 [M + Na]^+^ (calcd. for 401.1940). For the ^1^H and ^13^C NMR data, see Table 1.

X-ray crystallographic analysis of **1**: Colorless crystals of **1** were obtained from MeOH by slow evaporation. X-ray diffraction data were measured on an Agilent Gemini Ultra diffractometer (CuKα radiation, λ = 1.54178 Å) at 293 K. The structure was solved by direct methods (SHELXS-97) and refined using full-matrix least squares difference Fourier techniques. All non-hydrogen atoms were refined anisotropically, and all hydrogen atoms were placed in idealized positions and refined as riding atoms with the relative isotropic parameters. Monoclinic, C_21_H_30_O_6_, space group *P*2_1_, *a* = 10.9167 (4) Å, *b* = 7.5903 (2) Å, *c* = 12.2976 (4) Å, α = γ = 90.00, β = 109.063 (4), V = 803.78 (5) Å^3^, *Z* = 2, *D*_calcd_ = 1.305 g/cm^3^, μ = 0.775 mm^−1^, *F*(000) = 408, Flack = −0.23 (17). Crystal size: 0.41 × 0.38 × 0.15 mm^3^. Independent reflections: 2947 [*R*_int_ = 0.0207]. The final indices were *R_1_* = 0.0319, *wR_2_* = 0.0801 (*I* > *2*σ *(I)*).

### 3.4. Assays for Inhibitory Activity

All of the assays were carried out using 0.01 M KH_2_PO_4_/K_2_HPO_4_ buffer, pH 7.0, using a Shimadzu 2450 spectrophotometer. Enzyme solutions were prepared to give 2.0 units/mL in 2-mL aliquots. The assay medium contained phosphate buffer, pH 7.0 (950 µL), 10 µL of enzyme, 20 µL DMSO or inhibitor (dissolved in DMSO) and 20 µL of 0.01 M substrate (*p*-nitrophenyl (PNP) glycosides (3 mg/mL). The substrate was added to the assay medium containing enzyme and buffer with the inhibitor after 20 min of incubation time, and then, the increase in absorbance was measured at 400 nm for 1-min intervals at 37 °C. The percentage of inhibitory activity was calculated according to the equation: η (%) = ((B − S)/B) × 100% (B, the assay medium with DMSO; S, the assay medium with inhibitor). Values of IC_50_ were calculated according to the inhibitory activity curve. All measurements were done in triplicate from two independent experiments. The reported IC_50_ was the average value of two independent experiments.

## 4. Conclusions

*Epicoccum* sp. associated with *Apostichopus japonicus* was cultivated in liquid media, and a chemical investigation of cultures of this strain led to the isolation of one new isopimarane diterpene (**1**), together with two known compounds, 11-deoxydiaporthein A (**2**) and isopimara-8(14),15-diene (**3**). The absolute configuration of Compound **1** was deduced as 5*R*, 6*S*, 10*S*, 11*R*, 13*R* and 14*S* by a single-crystal X-ray diffraction experiment with CuKα radiation. All isolated compounds were evaluated for their α-glucosidase inhibitory activity, and both Compounds **1** and **2** exhibited α-glucosidase inhibitory activity with IC_50_ values of 4.6 ± 0.1 and 11.9 ± 0.4 μM, respectively. To our best knowledge, this was the first report on isopimarane diterpenes with α-glucosidase inhibitory activity. These results suggest that fungi associated with sea cucumber could be a good resource of anti-α-glucosidase agent.

## Supplementary Files

Supplementary File 1
