# Supplementary material for: Bioactive Isopimarane Diterpenes from the Fungus, Epicoccum sp. HS-1, Associated with Apostichopus japonicus
Source: Mar Drugs. 2015 Mar 2;13(3):1124–32. doi: 10.3390/md13031124 (PMC4377976; doi:10.3390/md13031124)
Supplement: Supplementary File 1 [file marinedrugs-13-01124-s001.pdf]

Supplementary Information

Figure S1. ^{1}H NMR spectrum of compound **1**

Figure S2. ^{13}C NMR and DEPT spectra of compound **1**

Figure S3. ^{1}H-^{1}H COSY spectrum of compound **1**

Figure S4. HMQC spectrum of compound **1**

Figure S5. HMBC spectrum of compound **1**

Figure S6. CD spectrum of compound **1** recorded in MeOH at ambient temperature

Figure S7. HRESITOFMS spectrum of compound **1**

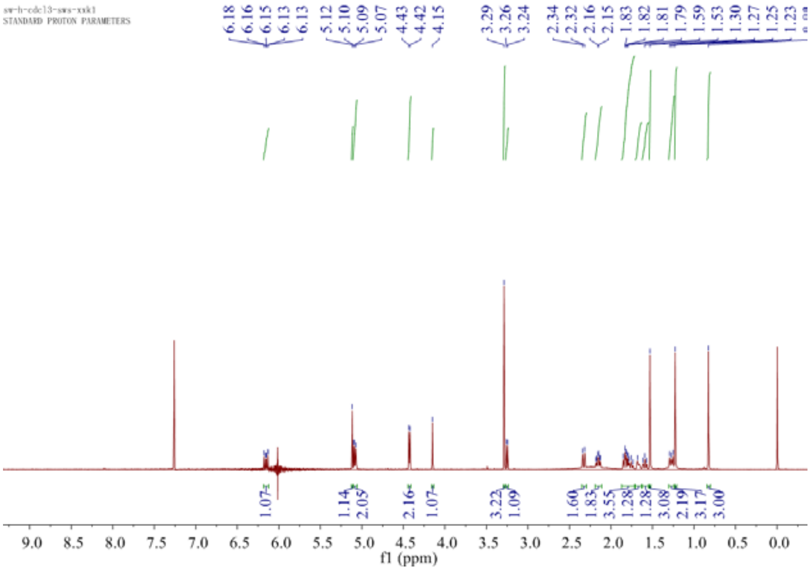

Figure S1. ^{1}H NMR spectrum of compound **1**.

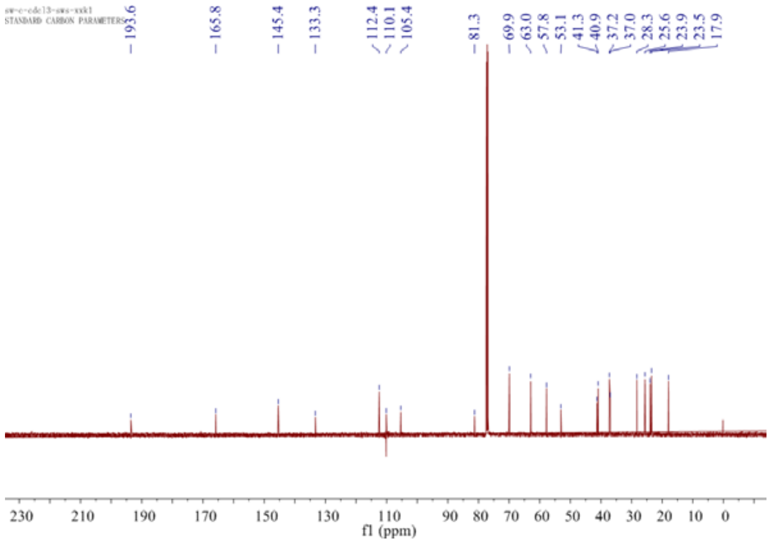

Figure S2. *Cont.*

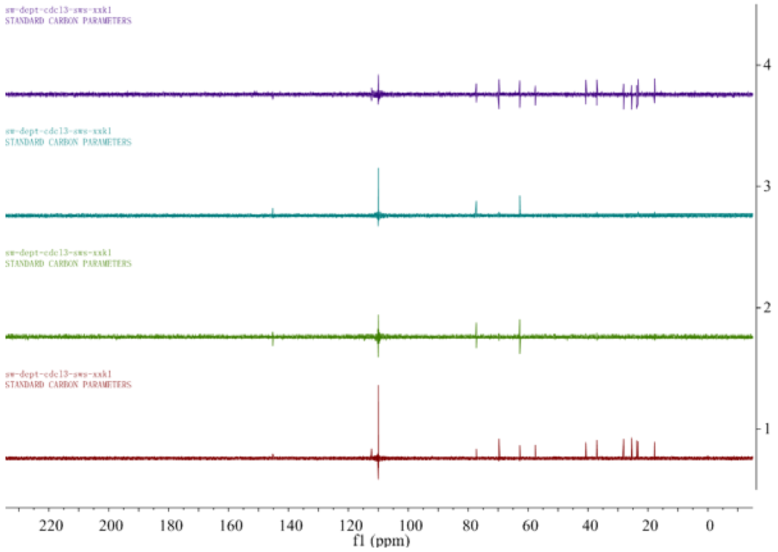

Figure S2. ^{13}C NMR and DEPT spectra of compound **1**.

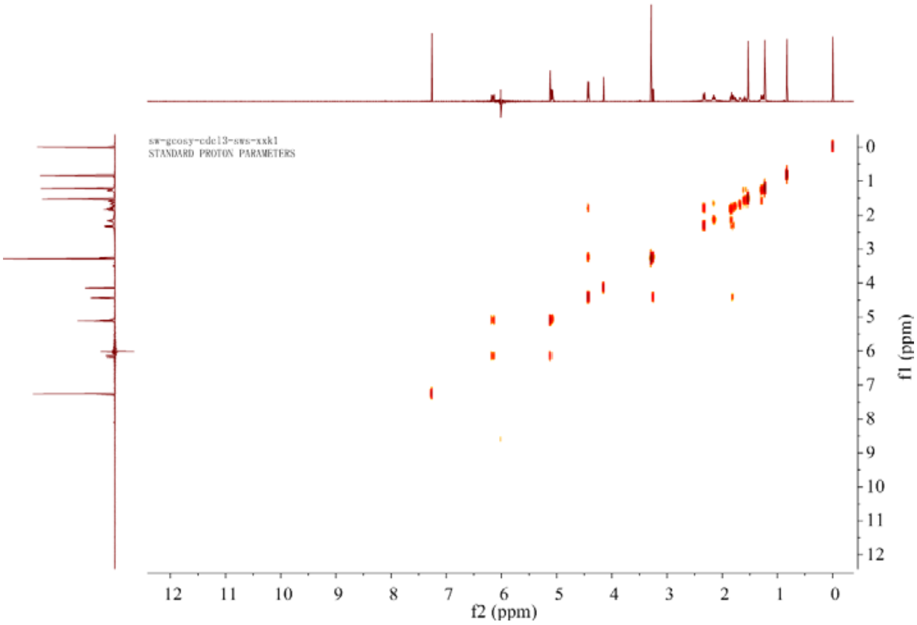

Figure S3. ^{1}H-^{1}H COSY spectrum of compound **1**.

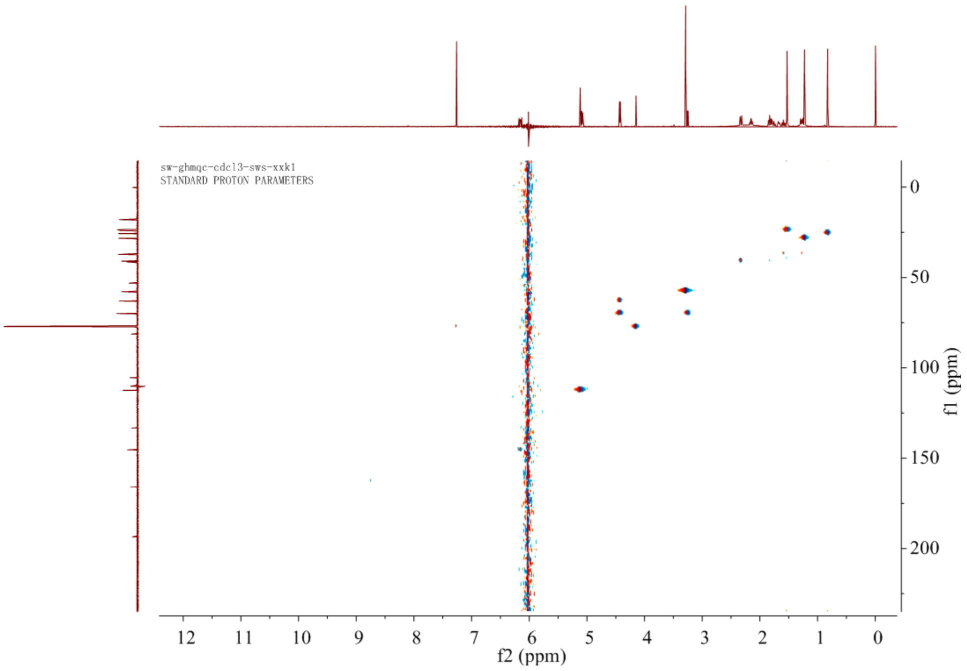

Figure S4. HMQC spectrum of compound **1**.

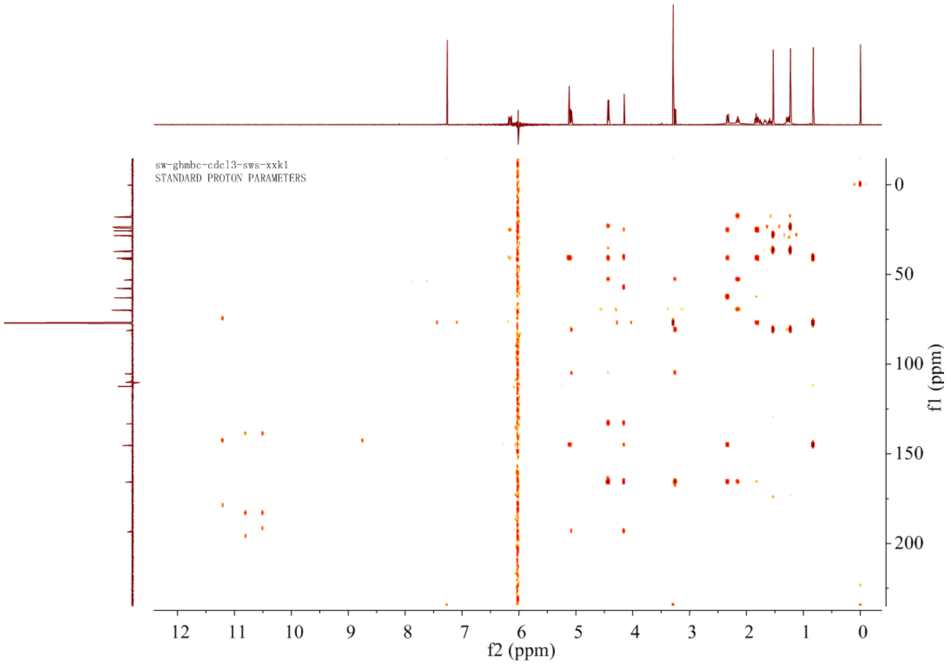

Figure S5. HMBC spectrum of compound **1**.

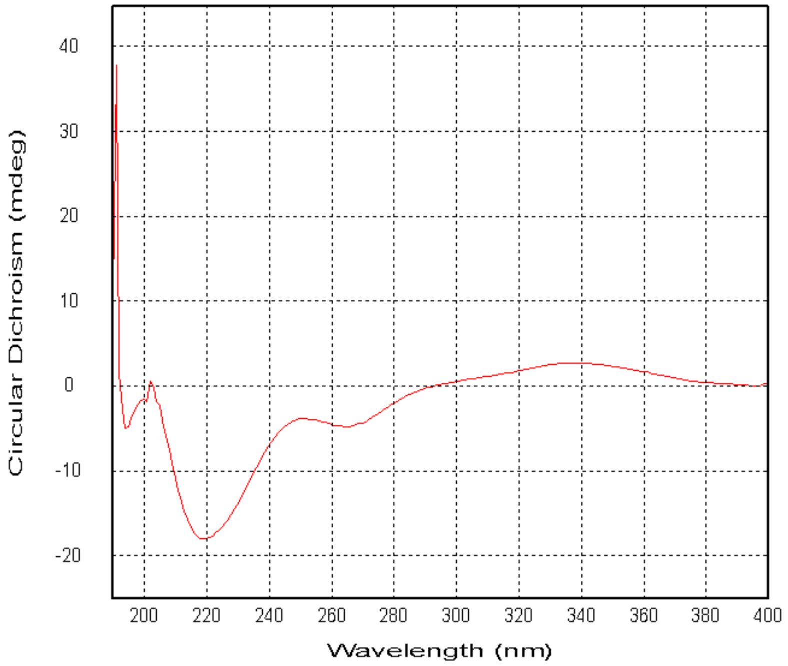

Figure S6. CD spectrum of compound **1** recorded in MeOH at ambient temperature.

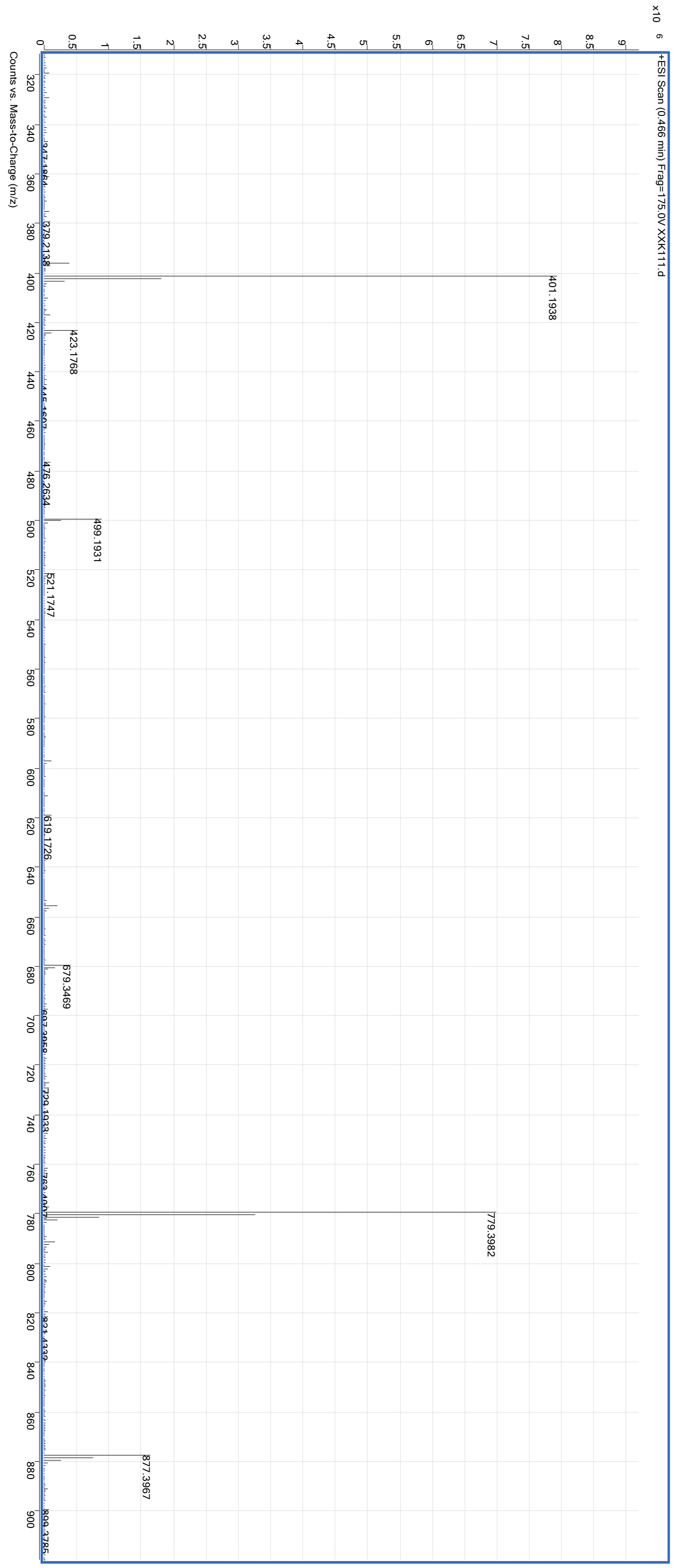

Figure S7. HR- ESI - TOF -MS spectrum of compound **1**.

© 2015 by the authors; licensee MDPI, Basel, Switzerland. This article is an open access article distributed under the terms and conditions of the Creative Commons Attribution license (http://creativecommons.org/licenses/by/4.0/).
